# Reactive Oxygen Species and Antioxidant System in Selected Skin Disorders

**DOI:** 10.21315/mjms2023.30.1.2

**Published:** 2023-02-28

**Authors:** Juliana Md Jaffri

**Affiliations:** Kulliyyah of Pharmacy, International Islamic University Malaysia, Pahang, Malaysia

**Keywords:** skin, oxidative stress, antioxidant, psoriasis, acne vulgaris, vitiligo, atopic dermatitis

## Abstract

The skin has a solid protective system that includes the stratum corneum as the primary barrier and a complete antioxidant defence system to maintain the skin’s normal redox homeostasis. The epidermal and dermal cells are continuously exposed to physiological levels of reactive oxygen species (ROS) originating from cellular metabolic activities. Environmental insults, such as ultraviolet (UV) rays and air pollutants, also generate ROS that can contribute to structural damage of the skin. The antioxidant defence system ensures that the ROS level remains within the safe limit. In certain skin disorders, oxidative stress plays an important role, and there is an established interplay between oxidative stress and inflammation in the development of the condition. Lower levels of skin antioxidants indicate that oxidative stress may mediate the pathogenesis of the disorder. Accordingly, the total antioxidant level was also found to be lower in individuals with skin disorders in individuals with normal skin conditions. This review attempts to summarise the skin oxidant sources and antioxidant system. In addition, both skin and total antioxidant status of individuals with psoriasis, acne vulgaris, vitiligo and atopic dermatitis (AD), as well as their associations with the progression of these disorders will be reviewed.

## Introduction

Skin is the largest organ of the human body and unlike most organs, its surface area is vulnerable to environmental insults. Consequently, skin is a target for the generation of reactive oxygen species (ROS) and oxidative stress. ROS play well established roles in the development of many diseases, including cancer, aging, atherosclerosis and cardiovascular diseases. Notorious for cellular damaging effects, ROS include singlet oxygen (^1^O_2_), superoxide anion (^•^O_2_^−^), hydroxyl radical (^•^HO) and the non-radical hydrogen peroxide (H_2_O_2_). Multiple endogenous sources of ROS have been proposed previously, including the mitochondria, peroxisomes, endoplasmic reticulum (ER), various oxidases, cytochrome P450 family, cyclooxygenases and lipoxygenases (1). Exogenous insults may also induce the production of ROS. Possible sources are ultraviolet (UV) radiation, xenobiotics and environmental stimulus, such as air pollutants.

Various cutaneous conditions have been associated with dysregulation of the antioxidant system and elevated levels of oxidative stress markers. Even though underlying mechanisms have not yet been elucidated, many studies have demonstrated that treatment with antioxidants improved the abovementioned conditions (2). In this review, different major endogenous and exogenous sources of ROS relevant to the skin will be discussed. Major changes in the antioxidant defence found in the skin and plasma that are linked to different skin disorders and conditions will also be highlighted.

### Cellular Sources of Reactive Oxygen Species

#### Mitochondria

ROS are continuously generated at a basal level as a by-product of normal cellular processes. In the mitochondria, 1%–5% consumption of oxygen (O_2_) results in the formation of ^•^O_2_^−^. The diminishing function of the cellular powerhouse increases ROS generation, elevating the risk of mitochondrial DNA damage and mutation. This vicious cycle progresses to extensive damage and, eventually, apoptosis (3). In chronic situations, such as during the aging process, skin structural changes will occur as the accumulated ROS damages dermal proteins and oxidises lipid membranes (4).

#### Peroxisome

The skin is active at metabolising lipids, which is one of the main components of the peroxisomes. β-oxidation of fatty acids occurs in the peroxisomes, particularly when the fatty acid chains are very long. This process is catalysed by acyl-coA oxidase and produces electrons from FADH_2_ that are transferred to O_2_, producing H_2_O_2_ as a by-product and energy, which is released as heat. In short, ROS accumulation is one of the outcomes of β-oxidation, which explains the vast amount of catalase (CAT) present in peroxisomes to degrade H_2_O_2_ (5).

#### Endoplasmic Reticulum

The folding of proteins in the ER is an oxidative process and is highly dependent on the oxidative potential of the ER lumen (6). The by-product of this bond formation is H_2_O_2_, which further increases the oxidative environment of the ER lumen. The presence of effective redox mechanisms, such as the glutathione/glutathione disulphide (GSH/GSSG) cycle, help maintain the redox homeostasis. However, in the event of an elevated protein folding load, evidence suggests that it may predispose the ER to oxidative stress (7). An unsuccessful cycle of disulphide bond formation could accumulate a substantial amount of H_2_O_2_ and cause a disruption in the balance of GSH/GSSG and compromise ER redox state (7).

#### Nicotinamide Adenine Dinucleotide Phosphate Oxidases

Nicotinamide adenine dinucleotide phosphate (NADPH) oxidases (NOX), found in virtually all tissues, including the skin, are notoriously linked to many chronic diseases due to their ROS generating behaviour (8). Recent findings have suggested that this may be a part of the full picture of NOX’s signalling features and outcome. Increasing recent evidence has demonstrated that ROS produced by NOX act as a cellular signal that maintains and re-establishes homeostasis during transient insults. This is an important cell survival pathway to promote stress resistance and longevity (9). However, when the stress results in irreversible cell injury, NOX may also convey signals that induce apoptosis (8). During persistent stimulation of NOX, overwhelming ROS production disrupts the sensitive redox balance of the cells, which, in turn, has been associated with a wide spectrum of chronic skin diseases, aging and the development of skin cancer (10).

### Environmental Sources of ROS

#### Ultraviolet Radiation

Solar UV radiation that reaches the earth can be divided into two different categories: UVA and UVB, with wavelengths between 320 nm–400 nm and 290 nm–320 nm, respectively (11). UVA accounts for up to 95% of UV radiation that reaches the earth. Its intensity remains relatively similar throughout the day and it can penetrate clouds and glass. It can penetrate both the epidermis and the dermis layers. Consequently, the skin is exposed to UVA more than it is to UVB throughout the day. UVB’s intensity is proportional to the amount of light received during the day, the highest being from 10 am to 4 pm daily, especially in countries near the equator, such as Malaysia. It penetrates the epidermis layer and may cause skin erythema on prolonged exposure. The resulting inflammation generates ROS, which directly damages skin protein, lipid and DNA.

Prolonged exposure to sunlight and UV radiation has been known to be detrimental to the skin. Skin contains photosensitisers that are excited when exposed to UV radiation, which results in the generation of free radicals or direct photochemical changes of various skin biomolecules, including flavin, melanin, porphyrins and nicotinamide (12). In the presence of excited photosensitisers, the absorbed energy can either be transferred to heat or cause covalent bonds to break to form photoproducts. The excited molecules may also react with other biomolecules, resulting in molecular changes. Free radicals may form in two different ways: i) Type I reaction, wherein the excited photosensitiser reacts directly with another biomolecule via an electron transfer to produce free radicals or stable reaction products and ii) Type II reaction, wherein the excited photosensitisers transfer their excess energy directly to O_2_, resulting in the formation of ^1^O_2_ (13). This powerful oxidant is capable of inducing cellular structure damages, including lipid peroxidation, formation of carbonylated protein and modification of DNA bases. Alternatively, ^•^ O_2_^−^ can also be generated by the transfer of an electron to ^1^O_2_.

#### Airborne Pollutants

Airborne pollutants are well recognised for contributing to ill health effects. According to the World Health Organization (WHO) Air Quality guidelines (14), there are four major air pollutants: i) particulate matter; ii) ozone; iii) nitrogen dioxide and iv) sulphur dioxide. These substances can increase skin oxidative stress through lipid peroxidation and, eventually, reducing the skin antioxidant status and impairing the skin barrier function. Ozone, for instance, was reported to cause lipid peroxidation and form protein carbonyl, which eventually increases skin wrinkling (15). A number of inflammatory skin diseases and increased risk for developing skin cancer have been associated with air pollutants through the regulation of oxidative stress and inflammatory cytokines (16).

### Antioxidants in the Skin

#### Enzymatic Antioxidants

**Figure 1:**
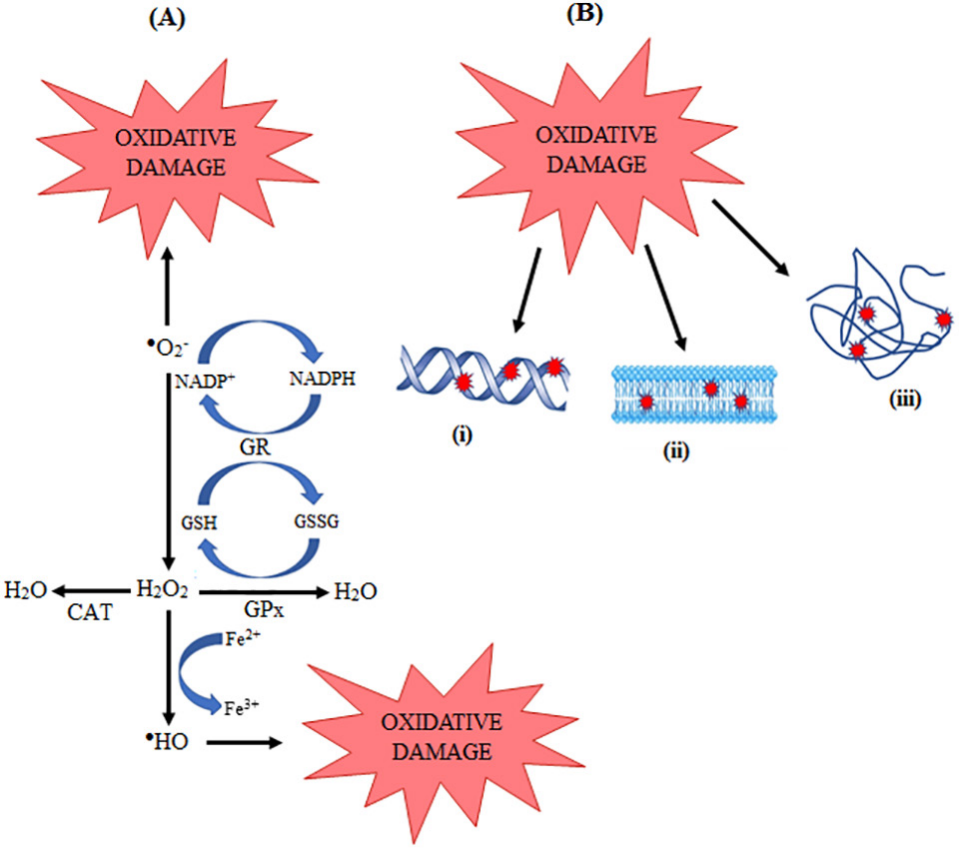
Antioxidant enzymes in the skin. SOD, GPx and CAT are found in the skin layers (A) to protect skin macromolecules from oxidative damage (B) which include: i) oxidative nucleotide damage, ii) lipid peroxidation and iii) protein carbonylation

The human body is equipped with a comprehensive antioxidant defence to combat the endless oxidative insult generated intrinsically or from environmental contaminants. This defence mechanism comprises enzymes, such as superoxide dismutase (SOD), glutathione peroxidase (GPx), CAT and glutathione reductase (GR) (Figure 1), and other molecules, including vitamin C, vitamin E and GSH. In addition, the carotenoid group of antioxidants, mainly lutein, lycopene, b-carotene and zeaxanthin, are also found in the skin layers. Being the largest organ, the skin content of endogenous antioxidants is expected to be in ample amount, which may be even greater than that of other organs. With the skin being continuously exposed to exogenous oxidative insults, there is a significant requirement for the great capacity of its antioxidant defence. The epidermis has been found to exhibit greater antioxidant capacity in comparison with the dermis layer (17). This greater capacity is essential, as being the outermost layer, the epidermis, particularly the stratum corneum, serves as the principal protective barrier of the body against the harsh environment.

Within the epidermis, SOD and CAT activities were found to gradually decrease towards the skin surface. Exposure of the skin to the sun leads to changes in different antioxidant enzyme activities. CAT activity has been shown to have reduced activities when the skin is more exposed to sunlight. In contrast, SOD activity remains unaffected by chronic sun exposure (18). This effect was found to be attributed to UVA exposure, while UVB appeared not to exhibit this effect. However, skin regained normal CAT activity within 4 weeks of the exposure (18).

### Superoxide Dismutase

The first line of antioxidant defence, particularly in the mitochondrial matrix, is the enzyme SOD (19). It scavenges ^•^O_2_^−^, which is the by-product of oxidative phosphorylation, and converts it into H_2_O_2_ and O_2_. This action is important to ameliorate the action of ^•^O_2_^−^, which, even though short-lived, may induce lipid peroxidation, collagen degradation and DNA damage. These eventually induce alterations in skin conditions, such as skin wrinkling and pigmentation, both of which are observed on photo-damaged skin (20).

Currently, three forms of SOD that contain different covalently bound substances (Cu, Zn and Mn) have been identified in the skin, including Cu/Zn-SOD, Mn-SOD and extracellular SOD (EC-SOD) (19). While Mn-SOD is compartmentalised in the mitochondria and Cu/Zn-SOD is located in the cytosol, EC-SOD is expressed by certain extracellular tissues, including the skin (19). SOD has been detected in the different skin layers, particularly the epidermis and the dermis layers. In the stratum corneum, the first 10 layers contain higher levels of SOD (18). The epidermal Cu/Zn-SOD level has been shown to be higher in younger females than in older females and males show slightly higher epidermal Cu/Zn-SOD level than females (21).

### Glutathione Peroxidase

This selenium-containing enzyme catalyses various lipid hydroperoxides and H_2_O_2_ at the expense of GSH (22). The end-product of this reaction is water. The dismutation of ^•^O_2_^−^ by SOD accumulates hydroperoxides and H_2_O_2,_ which are eventually eliminated by GPx, which is found in both the mitochondria and the cytosol. H_2_O_2_ accumulation increases the risk of Fenton reaction, which generates ^•^HO in the presence of transition metals (23).

GPx is expressed in tissues where SOD is detected, which includes the skin. The co-existence of SOD and GPx ensures that the hydroperoxides and H_2_O_2_ formed are rapidly detoxified to prevent the aggressive generation of ^•^HO and, hence, maintain homeostasis (24). Maintenance of the skin GPx level, along with those of SOD and GSH has been shown to reduce inflammation and promote healing of wounded skin (25). The role of GPx is highly dependent on the GSH/GSSG cycle via glutathione reductase in order to maintain the reduced GSH level and sustain normal cellular redox status (Figure 1).

### Catalase

Similar to GPx, CAT breaks down H_2_O_2_, but its activity is much higher compared to GPx (26). Much of cellular H_2_O_2_ is produced by the breakdown of ^•^O_2_^−^ by SOD. Increased concentration of cellular H_2_O_2_ has been known to cause oxidative stress (27), particularly in the skin, where environmental insults increases the risk of oxidative stress. The presence of significant amount of CAT in the epidermis protects the skin from the extensive damage of H_2_O_2_ generated by SOD or by environmental peroxides (28).

### Non-Enzymatic Antioxidants

#### Carotenoids

Carotenoids are a group of phytochemicals that occur naturally as organic pigments. Among the vast amount of carotenoid compounds found in nature, humans take up about 30 carotenoids with their diet. Carotenoids are well-known for their anti-oxidative properties, with a high capacity of quenching ^1^O_2_ and trap peroxyl radicals (29). Once ingested, carotenoids are absorbed by various tissues, including the skin, the level of which increases with increase in the intake of fruits and vegetables (30). The compounds that have been detected in the skin are beta-carotene (31), lycopene (32), zeaxanthin and lutein (33). These compounds protect the skin against the damaging effect of ^1^O_2_, which is produced in the skin after exposure to UV radiation (29).

#### Vitamin C

As with any other living tissue in the body, the skin also has specific requirement for vitamin C. Skin’s vitamin C level is, in fact, higher than that in the plasma, indicating its accumulation in the skin, particularly in the epidermis, where the concentration can reach fivefold higher than that in the dermis (34). It undoubtedly plays an important role in maintaining skin health. Vitamin C deficiency disturbs collagen formation, causing poor wound healing and subcutaneous bleeding due to loss of connective tissue morphology.

Another biological function of vitamin C is formation of the skin barrier that protects against permeation of various substances from the environment as well as transdermal epidermal water loss (35). Vitamin C, as a well-known antioxidant, also plays a role in increasing resistance of the skin-derived ROS. Along with antioxidant enzymes and other antioxidants, vitamin C contributes significantly in quenching skin-derived oxidants, thereby reducing the damaging effect on the skin cellular structures (36). Repeated and overwhelming generation of oxidants, including ROS, has been associated with many skin diseases, which will be discussed later.

#### Vitamin E

Vitamin E is a significant first line chain-breaking antioxidant localised in the lipid compartment of the skin with 87% of the total vitamin E in the epidermis consists of α-tocopherol (37). Its concentration in the epidermis is higher than that in the dermis by 90% (17), with a gradient of the highest level found in the deepest layer of stratum corneum (38) as well as in the sebaceous gland secretion (39). This probably is due to the direct exposure of epidermis to environmental insults. The consequences of short- and long-term exposure of skin to the hostile environment include erythema, skin thickening, wrinkling and increased risk of skin cancer. Peroxyl radicals formed during exposure to UV radiation or air borne chemicals react more efficiently with vitamin E than with polyunsaturated fatty acids, generating tocopheryl radicals. Vitamin C supports the antioxidant activity of vitamin E by converting the tocopheryl radical back to its reduced state, i.e. α-tocopherol (40). These mechanisms protect the lipid structure and materials of the skin.

### Antioxidant Status in Skin Disorders

#### Psoriasis

Psoriasis is a debilitating auto-immune disease that commonly affects the skin and the joints. The disease is characterised by the appearance of raised, red, scaling spots or skin lesions caused by abnormal epidermal differentiation and hyperproliferation, with an increase in the epidermal cell turnover rate and T-cell infiltration. Psoriasis patients also exhibit increased levels of proinflammatory cytokines, including IL-6, IL-1β, IL-17A and IL-22 (41). This is well correlated with excessive generation of ROS, both from endogenous and exogenous sources, resulting in increased oxidative stress (42). Increasing levels of protein oxidation markers, malondialdehyde (MDA), serum nitric oxide and end related products are associated with increasing severity of the psoriatic severity index (43, 44). This could partly be the result of lower total antioxidant status in these patients, specifically the decreased SOD and CAT activities (45, 46). Part of the inflammation process is the release of phagocytic ROS and reactive nitrogen species (RNS) generated by the NOX family of enzymes, which further exacerbate the oxidative stress in the skin (47). Leveraging extracellular SOD expression managed to prevent psoriasis development in mice, indicating that increased ROS plays an important role in the pathogenesis of psoriasis and that modulating the oxidative stress may be a potential pathway in controlling the disease (48). Higher levels of protein carbonylation were found in both fibroblast and the skin of psoriatic patients, both in the lesional and non-lesional part of the skin, indicating that oxidative damage is present, even before any inflammatory infiltrate (49). Subsequently, the fibroblast goes through changes that results in abnormal immune reactions leading to the onset of the disease and formation of lesions.

**Figure 2:**
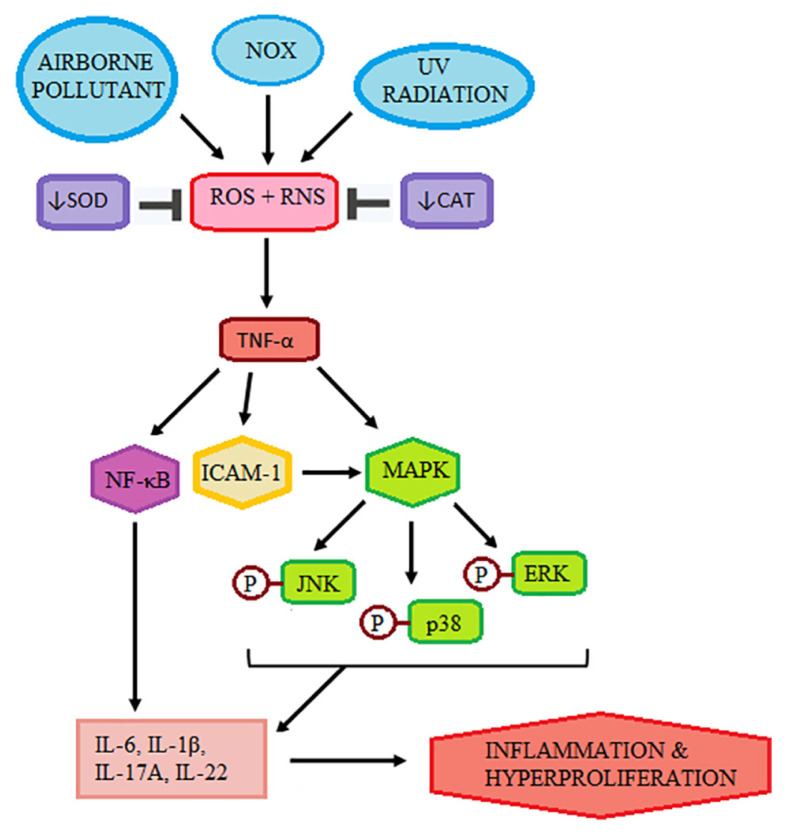
Accumulation of ROS and RNS from NOX, environmental stressors, and reduced antioxidant activity subsequently activate proinflammatory cytokines, leading to inflammation and epidermal hyperproliferation

Excessive ROS generation and inflammatory cytokines may initiate activation of tumour necrosis factor α (TNF-α), which, in turn, triggers the nuclear factor kappa B (NF-κB) and the cascade of mitogen-activated protein (MAP) kinase (MAPK) signalling pathway. In psoriasis, it was demonstrated that the intercellular adhesion molecule-1 (ICAM-1) and phosphorylation of c-Jun N-terminal kinase (JNK), the extracellular signal-regulated kinase ½ (ERK ½) and p38 MAPK were elevated, along with the activation of TNF-α (41). The MAPK pathways participate in important cellular functions, i.e. cell proliferation, differentiation and apoptosis (41). These signalling pathways were suggested to be dysregulated leading to an imbalance that could play an important role in the epidermal hyperproliferation of psoriasis. ICAM-1 is also known to trigger various pathways of inflammation, including through the stimulation of MAPK (50). Figure 2 summarises the proposed association between excessive ROS and psoriasis.

#### Acne Vulgaris

Acne vulgaris is a common skin condition highly associated with oily skin and affects men and women of all ages. It has a multifactorial pathogenesis and can exist in varying severity. In severe form, the lesions can be painful and result in uneven skin texture. Chronic inflammation is strongly associated with acne but the root cause of the inflammation is yet to be discovered. *Propionibacterium acnes (P. acnes)* found on the skin of patients with acne was found to be a more antigenic and pro-inflammatory strain, compared to that found on normal skin (51).

Active production of sebum is another characteristic of acne. Sebum is composed of triglycerides, squalene, wax esters and free fatty acids, and a small amount of cholesterol derivatives. Squalene is an unsaturated hydrocarbon that is prone to peroxidation, resulting in the formation of lipoperoxides, a known pro-inflammatory substance (52).

Follicular hyperkeratinisation was previously thought to be the outcome of inflammatory reaction and linked to the pathogenesis of acne (53, 54). Recent findings, however, showed that keratinocyte proliferation rate in both acne and non-acne hair follicles was identical (55, 56). Discrepancies may be due to the measurement of different markers of cell cycle progression and cell proliferation.

Sebum produced by the sebaceous gland empties onto the skin surface through the pore of the follicle. Excess sebum, together with keratinocytes and the hair, can obstruct the follicles and eventually form a plug. The mixture of keratinocytes and the sebum creates a favourable environment for the bacteria *P. acnes* to grow in the plugged follicles. *P. acnes* growth triggers the accumulation and activation of neutrophils. Phagocytosis causes the generation of ROS as part of the bacterial destruction mechanism and inflammatory response, which commonly injures the follicular epithelium. This is observed as swelling, redness, heat and pain.

**Figure 3:**
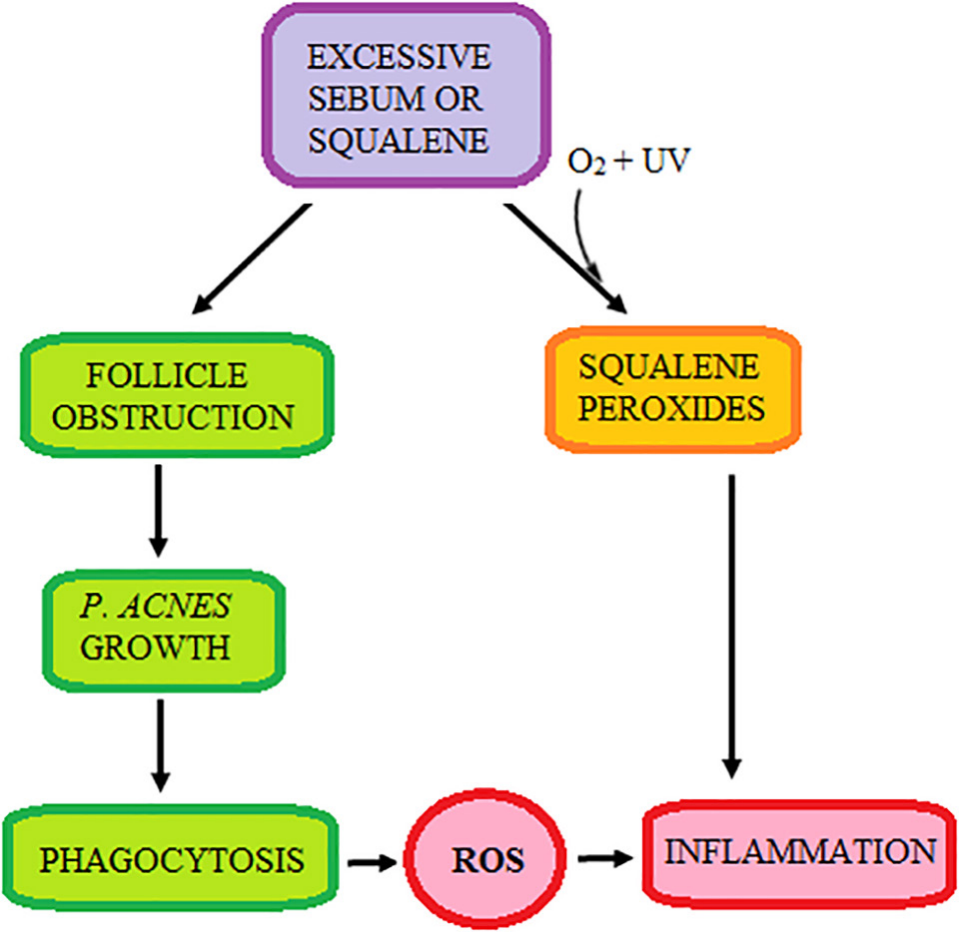
The development of inflammation in acne vulgaris via squalene peroxidation and excessive ROS

In normal skin and skin that is not exposed to excessive environmental insult that can trigger inflammation, the endogenous skin antioxidant defence is capable of coping with normal production of ROS in the skin. As acne is associated with increased inflammation and production of ROS, theoretically, skin antioxidant defence may be depleted. The level of GPx in skin specimens from lesions of acne vulgaris was found to be significantly lower compared to that from normal skin and it was inversely correlated with acne severity (57). Conversely, the level of MDA was significantly increased and positively correlated to acne severity. SOD, CAT and GSH were also found to decrease in the skin of acne patients (58). When the overall oxidant-antioxidant balance was studied in patients with acne, similar result was reported; the plasma levels of SOD, CAT, Vitamin E and zinc were decreased and plasma MDA level was increased significantly (59–61). There was also an inverse correlation between the plasma levels of CAT and MDA (62). These results indicated that acne is linked to both local and systemic oxidative stress, probably due to the inflammatory background of this disease. Figure 3 summarises the role of lipid peroxides and inflammation towards the skin redox balance in acne vulgaris.

#### Vitiligo

Vitiligo is a condition that affects the skin pigment—melanin. Worldwide prevalence of vitiligo ranges from 0.4% to 1.8%, with higher incidence within populations of colour (63). The disorder manifests clinically as gradual melanocyte autoimmune destruction from the epidermis and follicular reservoir and eventually results in significant depigmentation of the skin, most commonly observed as patches of uneven skin colour. The loss of melanocytes in vitiligo is unlikely due to a single dominant pathway, though there is evidence that, in many cases, oxidative stress is strongly associated with the disorder, with possible interaction with genetic, environment and immunological events. However, the contributory role of oxidative stress in the pathogenesis of vitiligo is still unclear.

The highly pro-oxidant state of the epidermis is thought to be part of the reason for melanocyte death. It was also found that epidermal CAT and GSH activities were lower in skin with vitiligo (16, 27, 64), whereas SOD activity was high (65). Due to the imbalance of enzyme levels, accumulation of intracellular H_2_O_2_ may result in the oxidative state of the epidermis, contributing to the pathogenesis of the segmental vitiligo through membrane peroxidation and, subsequently, melanocyte apoptosis (66, 67). It is possible that defective apoptosis can occur, and inefficient clearance of apoptotic cells results in protein derangement and accumulation of misfolded peptides. These deranged proteins may act as autoantigens that trigger autoimmunity (68). Recovery of the skin colour and eyelashes were also evident after epidermal H_2_O_2_ level is diminished (66). It was suggested that subtoxic level of H_2_O_2_ induced expression of IL-6 in the epidermal melanocytes, indicating that inflammation may play an significant role in the pathogenesis of this autoimmune disease (69). It was observed in vitro that oxidative stress induced translocation of the Ca^2+^-binding protein calreticulin (CRT) on melanocyte surface, which, in turn, induced the expression of pro-inflammatory cytokines, such as IL-6 and TNF-α. Elevated surface CRT levels resulted in decreased membrane CD_47_ expression, causing immunogenic melanocyte apoptosis (70). The role of H_2_O_2_ as one of the main culprits for the loss of melanocytes is still inconclusive. Interindividual variations in terms of skin phototypes as well as the different types of vitiligo could result in interestingly different findings.

**Figure 4:**
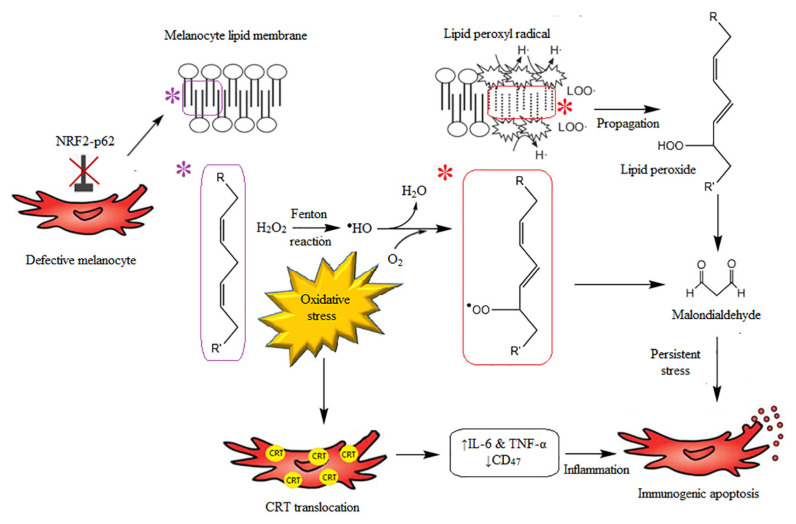
The interplay between defective melanocytes and oxidative stress, resulting in inflammation and immunogenic melanocyte apoptosis

Though both segmental and non-segmental vitiligo share similar inflammatory evolution and structural alteration, melanocyte loss in vitiligo patients may also be attributed to toxic accumulation of H_2_O_2_ per se or may be associated with alteration of the melanocyte sensitivity to H_2_O_2_ (71). In vitiligo, dysregulated autophagy has been demonstrated to increase the sensitivity of the melanocytes to H_2_O_2_ (72). The defect arises partly due to impairment in Nrf2-p62 pathway, which is essential for normal functioning of autophagy as a part of the cellular repair process to attenuate oxidative damage and maintain cell homeostasis (Figure 4) (72).

It is possible that the onset and progression of non-segmental vitiligo is not directly associated with the oxidative status of the skin. Overall systemic oxidative status may need to be taken into consideration. Increased levels of advanced oxidation protein products and advanced glycation end-products have been demonstrated in patients with non-segmental generalized vitiligo, and were found to be directly associated with the disease duration and extension, as well as the disease severity (73).

#### Atopic Dermatitis

The pathogenesis of atopic dermatitis (AD) remains unclear, although inflammatory response is well-known to play an important role in the development of AD, a chronic relapsing form of skin disorder. Its characteristics include dry skin caused by disturbed epidermal barrier and eruptions due to sensitisation to allergens originating from food and the environment, mediated by immunoglobulin E (IgE). These lead to itching, redness, cracking, weeping and eventually, crusting and scaling. It usually begins to affect during infancy and childhood, although it may also occur at any age.

**Figure 5:**
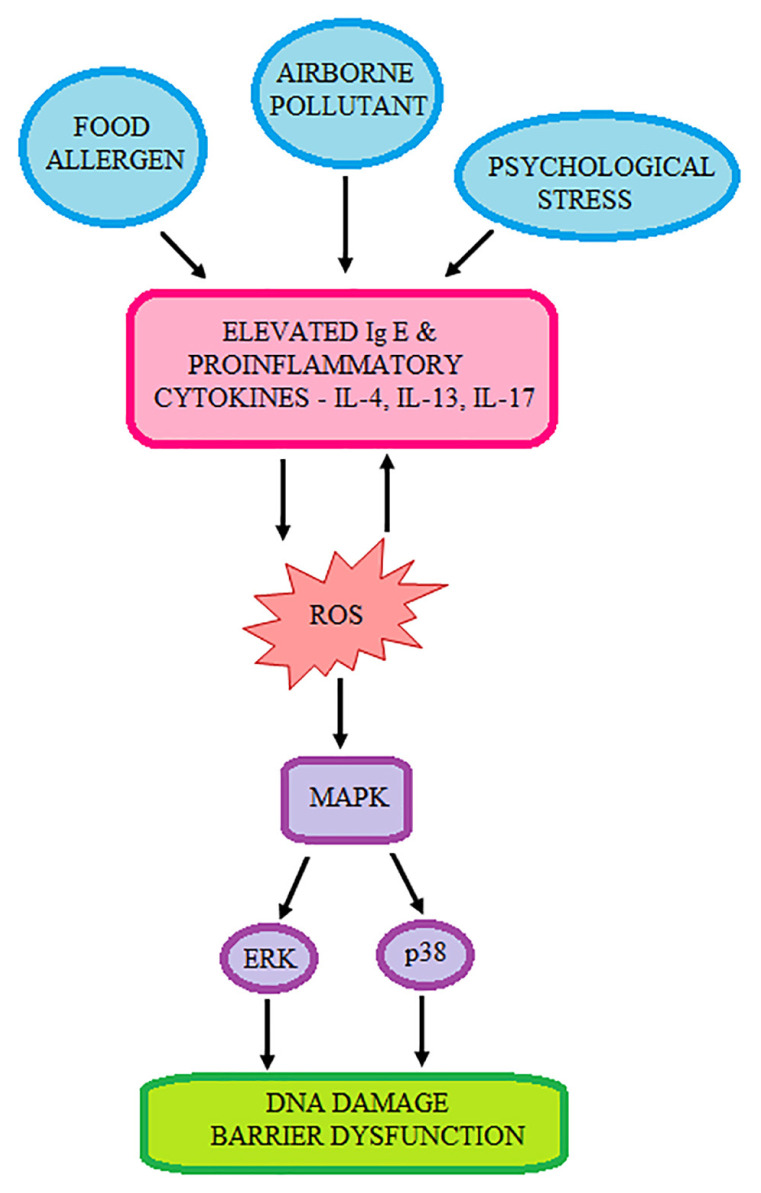
The inflammatory pathway of AD development, mediated by excessive ROS

Association between oxidative stress and AD has previously been suggested, with evidence showing increased lipid peroxidation that results in the decreased activities of SOD, CAT and GPx, and levels of non-enzyme antioxidants, i.e. GSH, and vitamins A, E and C (74). Interestingly, the lower plasma vitamin C levels did not result in reduced vitamin C in the lesional epidermis of AD patients, although the epidermal ceramide content reduced significantly (75). It was also observed that reduced level of vitamin A correlated with elevated levels of serum inflammatory markers, i.e. platelets and eosinophil, as well as higher red blood cell distribution width (76). An in vitro study conducted using normal human epidermal keratinocytes treated with IL-4, IL-13 and IL-17 resulted in high intracellular ROS level, which was translated as higher DNA damage (77). This may partly explain the correlation between elevated oxidative stress and inflammation causing diminished survival of keratinocytes with lesion formation in AD patients (Figure 5). Further increment of oxidative stress exacerbates AD through the involvement of MAP kinase signalling, particularly ERK and p38 signalling, with the latter considered to be pro-apoptotic (77). Elevated oxidative stress in this disorder may be attributed to environmental, physical or psychological stressors (78).

One of the risk factors of AD includes living in urban environment. Such environment is characterised by higher air pollution from vehicle exhaust fumes and industrialisation. Prolonged exposure to organic components, particulate matters and heavy metals of air pollutant may cause induce negative effect on the epidermal barrier due to their ability to generate ROS that can damage stratum corneum (79). Ozone, another air pollutant, is also notorious for its ill-effect on the skin. Exposure of the skin to ozone in polluted environment (0.8 ppm) results in depletion of epidermal α-tocopherol and induced cyclooxygenase 2, heme oxygenase I, indicating an increase in skin oxidative stress (80). As the exposure progresses, the epidermal barrier becomes disrupted and, as a consequent, intercellular penetration of particulate matter can occur, generating more ROS and causes dermal inflammation (81). Topical application of 100,000 IU SOD, combined with 4% plant extract (blackcurrant seed, sunflower seed and balloon vine extracts), seemed to alleviate AD after 30 days of twice a day application, indicating that ameliorating the antioxidative properties of the skin could help in AD amelioration (82).

## Conclusion

ROS are continuously being produced in the skin either due to cellular respiration or environmental factors. Nevertheless, it has a well-established antioxidant defence system to cope with physiological level of oxidation. Existing evidence points out that in certain skin disorders, there is an imbalance in cellular redox status and an association between this delicate balance and inflammation. Oxidative stress arises when there is prolonged extensive production of ROS, resulting in depletion of the natural cutaneous antioxidant system. This is evident in psoriasis, vitiligo and acne vulgaris. Total antioxidant status was found to be lower in psoriasis, vitiligo, acne vulgaris and AD patients. Although there is a significant amount of evidence that strongly suggests association between dysregulated skin redox homeostasis and certain skin disorders, studies on animal models as well as those that attempt to elucidate the molecular pathways are still limited. For many other skin diseases, substantial evidence is still scarce to fully understand the role of ROS as part of disease pathophysiology. This gap presents an opportunity for potential future studies to better understand the impact of cutaneous oxidative stress to enable strategizing the therapeutic use of antioxidants.
